# A high-throughput kinome screen reveals serum/glucocorticoid-regulated kinase 1 as a therapeutic target for NF2-deficient meningiomas

**DOI:** 10.18632/oncotarget.4858

**Published:** 2015-07-15

**Authors:** Roberta L. Beauchamp, Marianne F. James, Patrick A. DeSouza, Vilas Wagh, Wen-Ning Zhao, Justin T. Jordan, Anat Stemmer-Rachamimov, Scott R. Plotkin, James F. Gusella, Stephen J. Haggarty, Vijaya Ramesh

**Affiliations:** ^1^ Center for Human Genetic Research, Massachusetts General Hospital, Boston, MA, USA; ^2^ Department of Pathology, Massachusetts General Hospital, Boston, MA, USA; ^3^ Department of Neurology and Cancer Center, Massachusetts General Hospital, Boston, MA, USA; ^4^ Chemical Neurobiology Laboratory, Departments of Neurology and Psychiatry, Massachusetts General Hospital, Boston, MA, USA

**Keywords:** NF2, meningioma, mTOR signaling, SGK1, AZD2014

## Abstract

Meningiomas are the most common primary intracranial adult tumor. All Neurofibromatosis 2 (NF2)-associated meningiomas and ~60% of sporadic meningiomas show loss of NF2 tumor suppressor protein. There are no effective medical therapies for progressive and recurrent meningiomas. Our previous work demonstrated aberrant activation of mTORC1 signaling that led to ongoing clinical trials with rapamycin analogs for NF2 and sporadic meningioma patients. Here we performed a high-throughput kinome screen to identify kinases responsible for mTORC1 pathway activation in NF2-deficient meningioma cells. Among the emerging top candidates were the mTORC2-specific target serum/glucocorticoid-regulated kinase 1 (SGK1) and p21-activated kinase 1 (PAK1). In NF2-deficient meningioma cells, inhibition of SGK1 rescues mTORC1 activation, and SGK1 activation is sensitive to dual mTORC1/2 inhibitor AZD2014, but not to rapamycin. PAK1 inhibition also leads to attenuated mTORC1 but not mTORC2 signaling, suggesting that mTORC2/SGK1 and Rac1/PAK1 pathways are independently responsible for mTORC1 activation in NF2-deficient meningiomas. Using CRISPR-Cas9 genome editing, we generated isogenic human arachnoidal cell lines (ACs), the origin cell type for meningiomas, expressing or lacking NF2. NF2-null CRISPR ACs recapitulate the signaling of NF2-deficient meningioma cells. Interestingly, we observe increased *SGK1* transcription and protein expression in NF2-CRISPR ACs and in primary NF2-negative meningioma lines. Moreover, we demonstrate that the dual mTORC1/mTORC2 inhibitor, AZD2014 is superior to rapamycin and PAK inhibitor FRAX597 in blocking proliferation of meningioma cells. Importantly, AZD2014 is currently in use in several clinical trials of cancer. Therefore, we believe that AZD2014 may provide therapeutic advantage over rapalogs for recurrent and progressive meningiomas.

## INTRODUCTION

Neurofibromatosis 2 (NF2) is characterized by multiple nervous system tumors, including bilateral vestibular schwannomas, intracranial meningiomas, and spinal tumors such as schwannomas, meningiomas and ependymomas [1, 2]. Although most meningiomas are benign (WHO grade I), they often cause severe neurologic morbidity and mortality due to compression of the adjacent brain or spinal cord. Benign meningiomas have recurrence rates of up to 20% over 10 years. Twenty percent of meningiomas are classified as atypical (WHO grade II) or anaplastic (WHO grade III), and display more aggressive clinical behavior with faster growth and increased recurrence rates [3, 4]. The current standard of care for NF2-related meningiomas is maximal surgical resection with adjuvant radiation reserved for inoperable or progressive tumors, or those with aggressive features (e.g., WHO grades II or III). Meningiomas that progress despite surgery and radiation cause high morbidity, and the overall NF2 patient survival rate is ~38% at 20 years from diagnosis [5]. Therefore, effective non-invasive therapies are much needed for NF2-associated meningiomas and vestibular schwannomas, as well as their sporadic counterparts commonly seen in the general population.

The NF2 tumor suppressor protein merlin (NCBI definition, Neurofibromin 2/NF2*)* has been implicated in a wide range of mitogenic signaling pathways [6] in various cell types. However, the mechanism by which merlin/NF2 loss in human arachnoidal and Schwann cells results in meningiomas and schwannomas remains poorly understood. Employing patient-derived NF2-deficient meningioma cells and NF2 knockdown (shRNA) human arachnoidal cells, the cell of origin for meningiomas, we established that mammalian/mechanistic target of rapamycin complex 1 (mTORC1) is negatively regulated by merlin/NF2. mTORC1 is constitutively activated in NF2-associated schwannomas and meningiomas, and rapamycin was shown to block this mTORC1 activation [7, 8]. Subsequent studies carried out in mouse models reported that rapamycin suppressed the growth of meningiomas in a xenograft model [9] and delayed the growth of NF2-related Schwann cell tumorigenesis [10]. These studies led to clinical trials with mTORC1 inhibitor everolimus (RAD001), a rapamycin analog, for NF2 and sporadic meningiomas. Initial results from these clinical trials have been mixed, with one study reporting no shrinkage of vestibular schwannomas during everolimus treatment [11], and other studies reporting a delay in vestibular schwannoma growth during treatment [10, 12].

mTOR is an evolutionarily conserved serine/threonine kinase that regulates cell growth, proliferation and survival through two distinct functional complexes, mTORC1 and mTORC2, which signal to specific downstream targets [13, 14]. To further understand the role of merlin/NF2 in mTORC1 activation, we undertook an unbiased kinome screen in NF2-null meningioma cells. Here we report distinct activation of the mTORC2 target SGK1, detected by phosphorylation of its substrate NDRG1 (N-myc downstream-regulated gene1) in NF2-null human meningioma cells and NF2-deficient human arachnoidal cells, which remains insensitive to the mTORC1-specific inhibitor rapamycin. We further show that the selective mTOR kinase inhibitor AZD2014, targeting both mTORC1 and mTORC2, is more efficient than rapamycin in blocking proliferation of primary human meningioma cells and thus may hold promise as a more effective therapeutic option for NF2 patients.

## RESULTS

### High-throughput shRNA kinome screen reveals candidate kinases for constitutive mTORC1 activation in NF2-deficient cells

**Figure 1:**
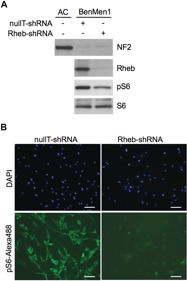
Constitutive mTORC1 activation in NF2-deficient meningioma cells is Rheb-dependent **A.** Immunoblotting of NF2-deficient meningioma cell line BenMen1 shows attenuated pS6 (mTORC1 readout) upon transduction of lentiviral Rheb shRNA compared to control (nullT) shRNA, under serum-deprived conditions. NF2 expression is shown compared to normal arachnoidal cell (AC) line. S6 serves as a control. **B.** Immunofluorescence staining of Benmen1 cells, transduced with lentiviral Rheb-shRNA (right panel), reveals significant attenuation of pS6-Alexa488 (green) staining compared to nullT-shRNA control (left panel). DAPI staining (blue) of cell nuclei is shown. Scale bar = 100μm.

**Figure 2:**
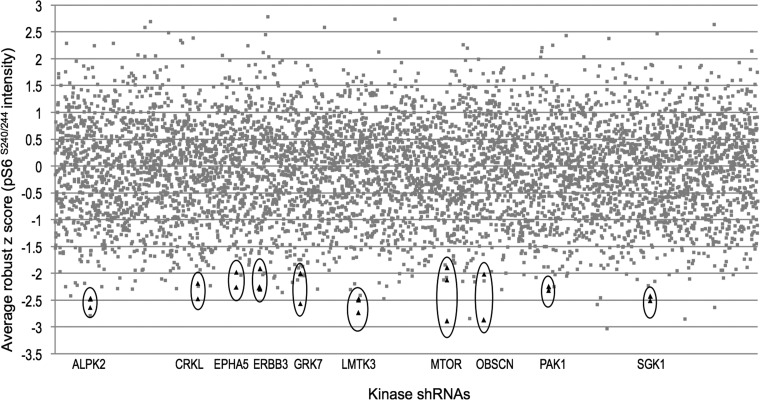
High-throughput kinome shRNA screen reveals kinases that rescue constitutive mTORC1 activation in NF2-deficient human meningioma cells Ten kinases with an average robust z score of < −1.8 were identified by reduced immunofluorescence staining of pS6 (S240/244) (mTORC1 readout) after infection of Ben-Men-1 cells with individual kinase shRNAs. Each spot represents an average of 3 replicates, with > 2 hairpins for each top hit shown (encircled black triangles).

We previously reported constitutive activation of mTORC1 signaling in NF2-deficient human arachnoidal cells (ACs), in primary meningioma cells and in NF2-associated tumors, meningiomas and schwannomas. We placed NF2 upstream of the tuberous sclerosis complex TSC1-TSC2 protein complex, which inhibits mTORC1 through TSC2 GAP activity toward the small GTPase Rheb. Our results showed that NF2 negatively regulates mTORC1 independent of PI3K/Akt and MEK/ERK pathways [7]. To further understand mTORC1 activation upon NF2 loss, we raised the question whether Rheb is required for this activation, and observed that suppression of Rheb rescues the constitutive activation of mTORC1 signaling by immunofluorescence and immunoblotting analyses (Figure 1), which confirmed that NF2 loss results in mTORC1 activation in a Rheb-dependent manner. Next we undertook an immunofluorescence-based, high-throughput kinome screen to identify kinases which, when suppressed, leads to decreased pathway activation using phosphorylated ribosomal S6 protein S240/244 (pS6) as a readout (assessed by decreased pS6 staining intensity). The primary screen was carried out in triplicate in the NF2-negative benign meningioma cell line Ben-Men-1 [15], using a high-titer lentiviral kinome shRNA library developed by The RNAi Consortium (TRC; Broad Institute/MIT, Cambridge, MA). Top hit calling was performed using robust z scoring methodology that is frequently employed in high-throughput RNAi screens to identify positives [16]. A list of top hit candidates emerged using the following criteria: 1) an infection efficiency > 60%, 2) two or more independent hairpins for an individual kinase displaying a robust z score < −1.8 (representing a reduction in pS6 staining intensity by >50% in our screen) observed in 3 replicates, and 3) no significant decrease in nuclei number to ensure that decreased pS6 was not due to decreased cell number. A secondary screen of independently packaged and assembled shRNA lentivirus (5 hairpins/candidate) for top hits in Ben-Men-1 cells confirmed the primary screen candidates. As predicted, mTOR emerged as a significant kinase with an average robust z score of −2.30 for 3 independent shRNA clones, thus showing proof-of-concept for the screen. Interestingly, two top kinases to emerge were serum/glucocorticoid-regulated kinase 1 (SGK1) and p21 protein-activated kinase 1 (PAK1). SGK1 and PAK1 were each identified by 2 independent shRNA clones in triplicate with robust z scores of −2.47 and −2.28, respectively (Figure 2, Table 1).

**Table 1:** Top candidate kinases identified in the high-throughput shRNA kinome screen

Gene ID	# shRNAs	robust z score^2^	Protein description
*LMTK3*	3	−2.57	lemur tyrosine kinase 3
*SGK1*	2	−2.47	serum/glucocorticoid-regulated kinase 1
*ALPK2*	2	−2.47	alpha kinase-2
*OBSCN*	2	−2.51	obscurin, cytoskeletal calmodulin and titin-interacting Rho-GEF
*CRKL*	2	−2.33	oncogene CRK-like; v-CRK avian sarcoma virus CT10-homolog-like
*MTOR*	3	−2.30	mammalian/mechanistic target of rapamycin
*PAK1*	2	−2.28	p21 protein-activated kinase 1
*GRK7*	2	−2.20	G protein coupled receptor kinase 7
*ERBB3*	3	−2.17	V-Erb-B2 avian erythroblastic leukemia viral oncogene homolog 3
*EPHA5*	2	−2.12	ephrin receptor EphA5
*RHEB*^1^	n/a	−2.00	Rheb-GTPase, shRNA control
*nullT*^1^	n/a	0.49	non-hairpin forming negative control
*GFP*^1^	n/a	0.12	negative hairpin control

1 positive and negative controls used on every plate

2 average robust z score from 3 replicates of each candidate hairpins

SGK1 is one of three primary downstream AGC kinase targets of mTORC2, which also include Akt and PKC-α [17-19]. Previously Huang *et al.* demonstrated that *Tsc2*-deficient mouse embryonic fibroblasts (MEFs) with constitutive activation of mTORC1 revealed inhibition of mTORC2 activity with attenuated phosphorylation of all three targets Akt, PKC-α and SGK1, using the effector pNDRG1 as a biomarker for SGK1 activation [20]. In contrast, we recently demonstrated that in NF2-shRNA suppressed human ACs and Schwann cells (SCs), while like *Tsc2^−/−^* MEFS show attenuated pAkt and pPKC-α, the mTORC2 target SGK1/pNDRG1 displays robust constitutive activation [21]. Regarding PAK1, NF2 protein is known to negatively regulate Rac1 signaling via inhibition of PAK1, and it has been demonstrated that loss of Nf2 expression leads to activation of Pak1 in rat Schwann cells [22, 23]. In addition, PAK1 activity is upregulated in NF2 patient-derived schwannomas [24]. Moreover, chemical compounds targeting PAK1 have been suggested as a potential therapeutic for NF2 [25, 26]. Given our recent observation of mTORC2-SGK1/pNDRG1 activation in NF2-deficient cells [21], as well as reports associating NF2 with Rac1/PAK1 signaling, we chose to follow up and expand our studies of these two candidate kinases.

### NF2-deficient cells show activation of SGK1/pNDRG1, and SGK1 inhibition rescues constitutive mTORC1/pS6 activation

**Figure 3:**
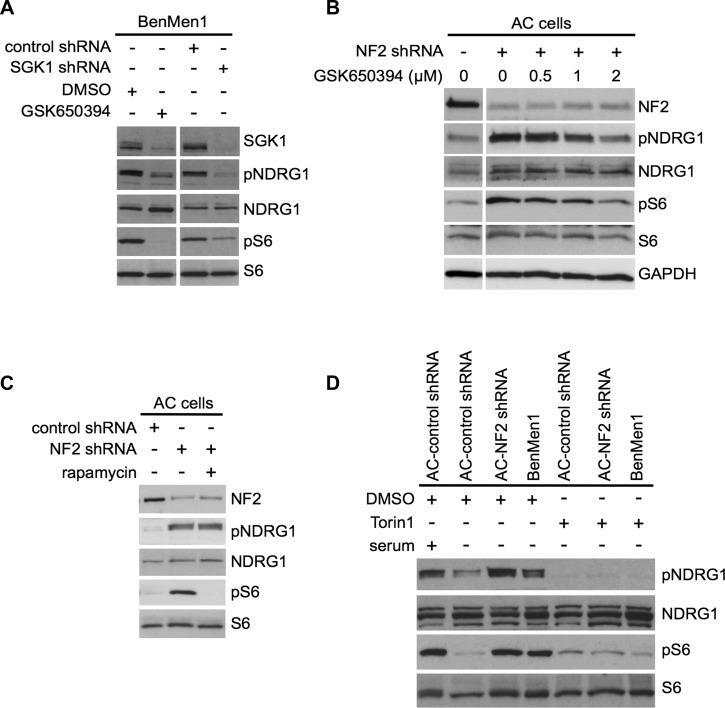
SGK1 regulates mTORC1 signaling in an mTORC2-dependent manner **A.** Immunoblot analysis with indicated antibodies of Ben-Men-1 cells treated with SGK1 inhibitor GSK650394 (2 μM, 18h; left panel) or SGK1-suppressed (shRNA, right panel) shows decreased SGK1 levels as well as attenuated NDRG1 (T346) phosphorylation (pNDRG1) and S6 (S240/244) phosphorylation (pS6) compared to DMSO treated or control shRNA, respectively. **B.** Immunoblotting with indicated antibodies of NF2-suppressed (shRNA) human arachnoidal cells (AC cells) treated with GSK650394 (0.5 - 2 μM) compared to DMSO (0) shows dose-dependent attenuation of constitutive pNDRG1 and pS6. Left panel, DMSO treated control ACs. **C.** Rapamycin treatment (20 nM, 2 h) of NF2 shRNA transduced ACs shows attenuation of pS6 but not pNDRG1 (SGK1 readout) compared to DMSO alone. **D.** In NF2-shRNA ACs or Ben-Men-1 cells, constitutive activation of both pNDRG1 and pS6 are attenuated upon treatment of with dual mTORC1/2 inhibitor Torin1 (250 nM, 1 h), compared to DMSO alone. NF2 expression levels are shown. NDRG1, S6 and GAPDH serve as controls.

Of the two independent SGK1-shRNA clones that rescued pS6 activation in our screen, we performed large-scale packaging of 1 clone to carry out further studies. Inhibition of SGK1 was performed using lentiviral SGK1-shRNA infection in Ben-Men-1 cells, or with the SGK1 inhibitor GSK650394 in both Ben-Men-1 cells and NF2-shRNA ACs. Both of these showed attenuation of mTORC1 signaling (pS6 readout) and downregulation of pNDRG1, a specific readout for SGK1 activation (Figure 3A and 3B). Importantly, while many recent studies demonstrate SGK1 as a downstream target of mTORC2 signaling, SGK1 has also been reported downstream of mTORC1 in some cell types [27]. We tested whether the observed constitutive SGK1/pNDRG1 activation was blocked by either mTORC1 inhibition alone or dual inhibition of mTORC1/mTORC2 signaling. We treated NF2-shRNA ACs and Ben-Men-1 cells with the allosteric mTORC1 inhibitor, rapamycin, or an ATP-competitive dual mTORC1/2 kinase inhibitor, Torin1. In both cellular models, we observed downregulation of SGK1/pNDRG1 with Torin1 treatment; however, pNDRG1 was not rescued in NF2-suppressed ACs using rapamycin (Figure 3C compared to 3D). This suggests that SGK1 activation is dependent on mTORC2 and independent of mTORC1.

### Inhibition of PAK1 rescues constitutive mTORC1 signaling but not mTORC2-SGK1/pNDRG1 in NF2-deficient cells

**Figure 4:**
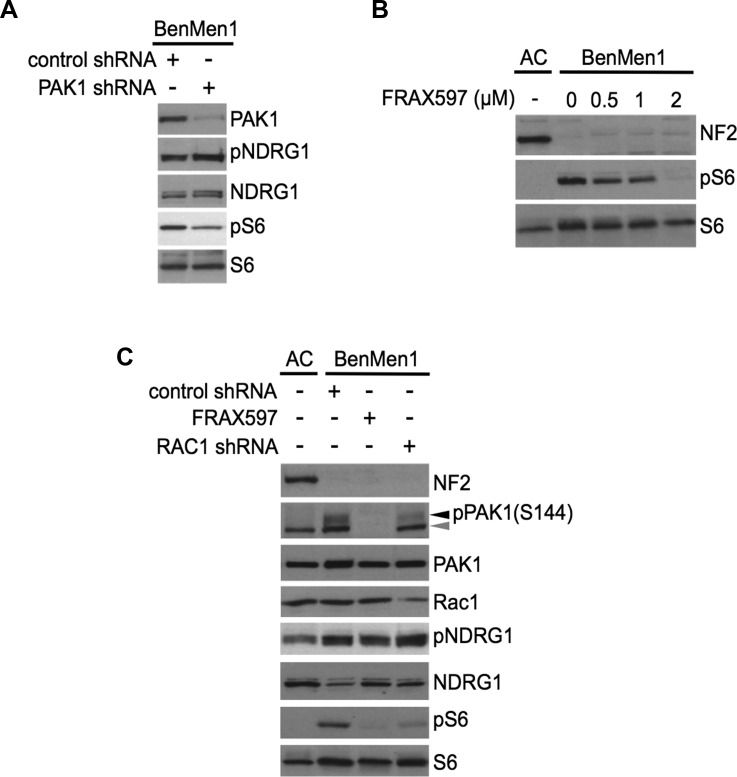
PAK1 regulates mTORC1 signaling independent of mTORC2-SGK1 **A.** Immunoblotting of PAK1 shRNA Ben-Men-1 cells with indicated antibodies reveals that PAK1-suppression attenuates pS6 with no change in pNDRG1 levels compared to control shRNA. **B.** Immunoblot analysis of Ben-Men-1 cells treated with the group I PAK inhibitor FRAX597 shows a dose-dependent decrease in constitutive pS6 compared to DMSO (0). **C.** Immunoblot analysis with indicated antibodies Ben-Men-1 cells treated with FRAX597 (2 μM, 2h), or RAC1 shRNA reveals attenuation of both PAK1 (S144) phosphorylation (pPAK1, black arrowhead) and pS6, but no inhibition of pNDRG1 compared to control shRNA. Antibody for pPAK1 (S144) also detects pPAK2 (S141, gray arrowhead). NF2-expressing ACs serve as a control **B.** and **C.**. All experiments were carried out under serum-deprived conditions unless otherwise noted.

In our high-throughput screen we identified two independent shRNA clones targeting PAK1 that rescued the constitutive mTORC1 activation in Ben-Men-1 cells (Table 1), and we performed large-scale packaging of 1 clone to carry out further studies. Inhibition of PAK1 using lentiviral PAK1-shRNA infection or treatment using the group I PAK inhibitor FRAX597 in Ben-Men-1 cells showed attenuation of pS6 (Figure 4A and 4B). FRAX597 treatment resulted in a dose-dependent decrease of pS6 and downregulation of pPAK1 S144 (Figure 4B and 4C). We also carried out shRNA suppression of RAC1 which also attenuated mTORC1/pS6; however, PAK1-shRNA, RAC1-shRNA and FRAX597 inhibitor treatment were unable to rescue constitutive activation of SGK1/pNDRG1, suggesting PAK1 and RAC1 are not required for activation of SGK1/pNDRG1, and may play a distinct role for constitutive mTORC1 activation in these cells (Figure 4A and 4C).

### NF2-null CRISPR ACs shows activation of mTORC1 and mTORC2/SGK1 signaling

**Figure 5:**
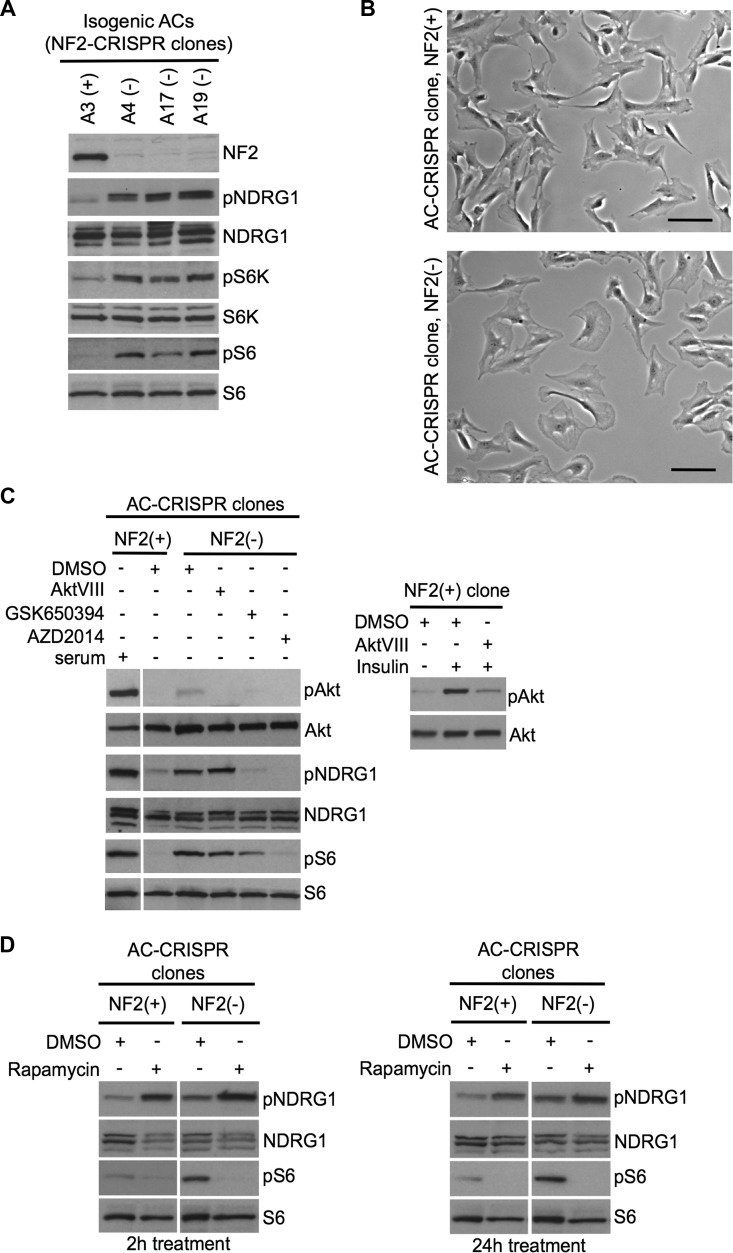
Inactivation of NF2 using CRISPR-Cas9 genome editing in human arachnoidal cells recapitulates enlarged cell morphology and signaling signatures of NF2-deficient human meningioma cells **A.** Immunoblotting with indicated antibodies of NF2-null (−) AC-CRISPR clone compared with NF2-expressing (+) AC-CRISPR clones demonstrates loss of NF2 expression, increased p70S6K (T389) phosphorylation (pS6K) and pS6 (mTORC1 readouts), and increased pNDRG1 (SGK1 readout) compared with NF2-expressing (+) clone. S6K, S6 and NDRG1 serve as controls. **B.** Representative bright-field images show enlarged cell morphology in NF2-null (A17) AC-CRISPR clone (right) compared to NF2-expressing (A3) clone (left). Scale bar = 100 μm. **C.** Left panel shows immunoblot analysis with indicated antibodies of an NF2(−) compared to NF2(+) AC-CRISPR clones treated with GSK650394 (2μM, 18h), dual mTOC1/2 kinase inhibitor AZD2014 (300 nM, 2h), AktVIII (1 μM, 2h) inhibitors or DMSO alone. As a control for Akt inhibition, AktVIII treatment of insulin-stimulated NF2(+) AC-CRISPR clone is shown (right panel). **D.** Immunoblotting with indicated antibodies of NF2(+) and NF2(−) AC-CRISPR clones treated with rapamycin (20nM) or DMSO for 2h (left panel) and 24h (right panel) shows an increase in pNDRG1 after rapamycin treatment.

NF2-shRNA knockdown ACs show significant reduction, but not total absence of NF2; therefore, we employed CRISPR-Cas9 genome editing using a single guide RNA targeting the human *NF2* exon 8 [28] in AC007-hTERT. Genotyping of selected single clones revealed 6 clones retaining wildtype (WT) sequence for *NF2* exon 8, 3 clones with homozygous *NF2* exon 8 in/del mutations, and 3 clones with compound heterozygous *NF2* exon 8 in/dels with distinct mutations on each allele, where all in/del mutations predicted putative premature stop codons. We chose independent clones of NF2-expressing ACs (clones A2(+) and A3(+), WT) and NF2-null CRISPR ACs (clones A4(−) and A17(−), compound het; and A19(−), homozygous) (Table 2) and tested the direct effects of NF2 loss versus NF2 expression in these clones. All three NF2-null AC-CRISPR clones showed constitutive activation of mTORC1 signaling with enlarged cell morphology consistent with our earlier study with NF2-shRNA in these cells [7], as well as activation of SGK1 as detected by pNDRG1 compared to NF2-expressing isogenic clones (Figure 5A and 5B). Consistent with our kinome screen results, SGK1-shRNA and SGK1 inhibitor GSK650394, as well as the dual mTORC1/2 kinase inhibitor AZD2014, inhibited pNDRG1 and pS6 in NF2-null CRISPR ACs under serum-deprived conditions (Figure 5C, left). In contrast, the Akt inhibitor AktVIII, while inhibiting insulin-stimulated pAkt S473 in NF2-expressing ACs (Figure 5C, right), showed no effect on pNDRG1 or pS6 level in NF2-null CRISPR ACs (Figure 5C, left). Akt activation was not observed in NF2-null CRISPR cells, confirming our previous reports [7, 21] that Akt activation is not responsible for constitutive mTORC1 activation in NF2 target cell types. These data indicate that mTORC1 activation in NF2-null AC-CRISPR cells, at least in part, is dependent on a novel mechanism involving distinct activation of mTORC2-SGK1 signaling that is independent of mTORC2-Akt signaling. Further, we examined whether rapamycin was capable of activating mTORC2-SGK1 in our cellular model, which is a known concern of rapamycin treatment through relieving feedback mechanisms toward mTORC2-Akt signaling [18]. Treatment with rapamycin for 2h and 24h inhibited mTORC1 signaling as measured by decreased pS6, however, mTORC2-SGK1 signaling, detected by pNDRG1, concomitantly increased in both the NF2-expressing and NF2-null AC-CRISPR cells grown under full serum conditions (Figure 5D) confirming that mTORC2-SGK1 signaling is activated by rapamycin. Taken together, these results suggest that dual inhibition of mTORC1 and mTORC2 could be more effective in NF2-deficient cells.

**Table 2:** Isogenic, clonally-derived lines generated from an immortalized arachnoid cell line using CRISPR-Cas9 genome editing

clone ID^1^	NF2 expression	NF2 (ex8) mutation^2^
A2	+	wildtype
A3	+	wildtype
A4	−	*compound heterozygous alleles* 1) 787del23bp, 263fs > 274X 2) 804insC, 269fs > 275X
A17	−	*compound heterozygous alleles* 1) 795del8bp, 265fs > 274X 2) 802insT, 268fs > 274X
A19	−	*homozygous* 787del35bp, E8/I8

1 ID numbers for NF2-expressing (+) and NF2-null (−) clones.

2 genotype for individual clones.

### NF2 loss in human ACs and meningioma cells leads to increased expression of SGK1

**Figure 6:**
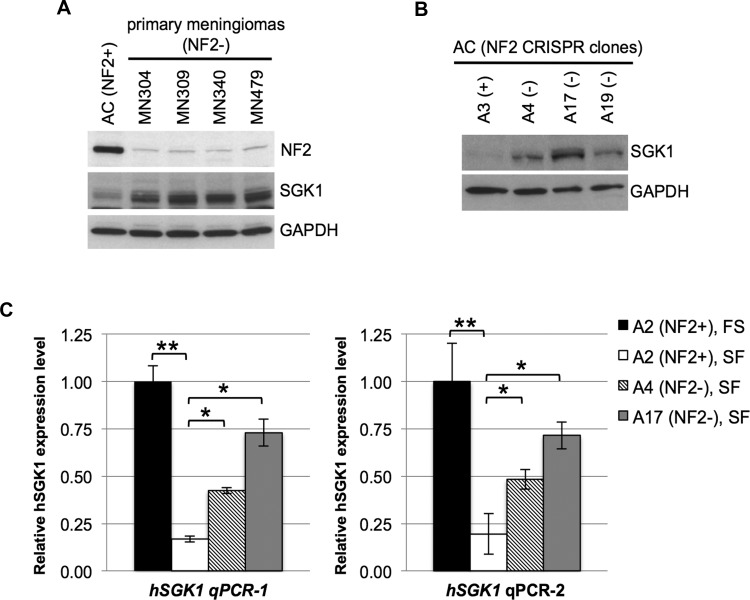
NF2-deficiency leads to increased expression of SGK1 **A.** and **B.** Immunoblot of patient-derived NF2-deficient primary meningiomas **A.** and NF2-null (A4, A17 and A19) AC-CRISPR clones **B.** shows increased SGK1 protein expression compared to normal (NF2+) ACs or NF2-expressing (A3) AC clone, respectively. NF2 expression is shown **A.** and GAPDH serves as a control **A** and **B.**. **C.** Quantitation of real-time RT-PCR of human *SGK1* (*hSGK1*) using 2 independent primer sets (*hSGK1* qPCR-1 and *hSGK1* qPCR-2) shows decreased SGK1 expression in serum-deprived NF2-expressing (A2, SF) AC-CRISPR clone compared to full serum (FS) conditions. Furthermore, under serum-deprived conditions (SF), isogenic NF2-null (A4 and A17) clones reveal a statistically significant increase in *SGK1* expression compared to NF2-expressing (A2) clone. Real-time PCR was carried out in triplicate for each primer set (3 independent experiments). Data are presented as mean +/− SD (*p < 0.05, **p < 0.005). FS, full serum conditions; SF, serum-free conditions.

Activation and expression of SGK1 are tightly controlled in response to serum stimulation and glucocorticoids, and increased expression is regulated at the transcriptional and post-translational levels [29]. Further, overexpression of SGK1 has been reported in multiple cancers, including breast cancer [30, 31]. Therefore we examined whether NF2 loss leads to increased expression of SGK1. Immunoblotting of 4 unrelated patient-derived primary NF2-deficient meningioma (MN) cell lines, as well as 3 NF2-null AC-CRISPR clones revealed increased SGK1 levels compared to control ACs (Figure 6A and 6B). Next we tested *SGK1* expression at the transcriptional level using 2 independent primer sets targeting human *SGK1* in isogenic NF2-CRISPR ACs. As expected, quantitative RT-PCR analysis of the NF2-expressing A2(+) clone showed decreased *SGK1* expression under serum-deprived conditions compared to full-serum conditions (Figure 6C). The NF2-expressing A3(+) clone also showed identical results (data not shown). Interestingly, under serum-deprived conditions, NF2-null AC-CRISPR clones A4(−) and A17(−) revealed 2.5 - 4 fold increased expression in *SGK1* compared to A2(+) (Figure 6C). Taken together, our results demonstrate that NF2 loss in ACs leads to elevated *SGK1* expression at the transcriptional level, which may partly explain the elevated SGK1/pNDRG1 signaling in these cells.

### Dual mTORC1/mTORC2 inhibitor AZD2014 is more effective than rapamycin or FRAX597 in decreasing cell viability in NF2-deficient meningioma cells

**Figure 7:**
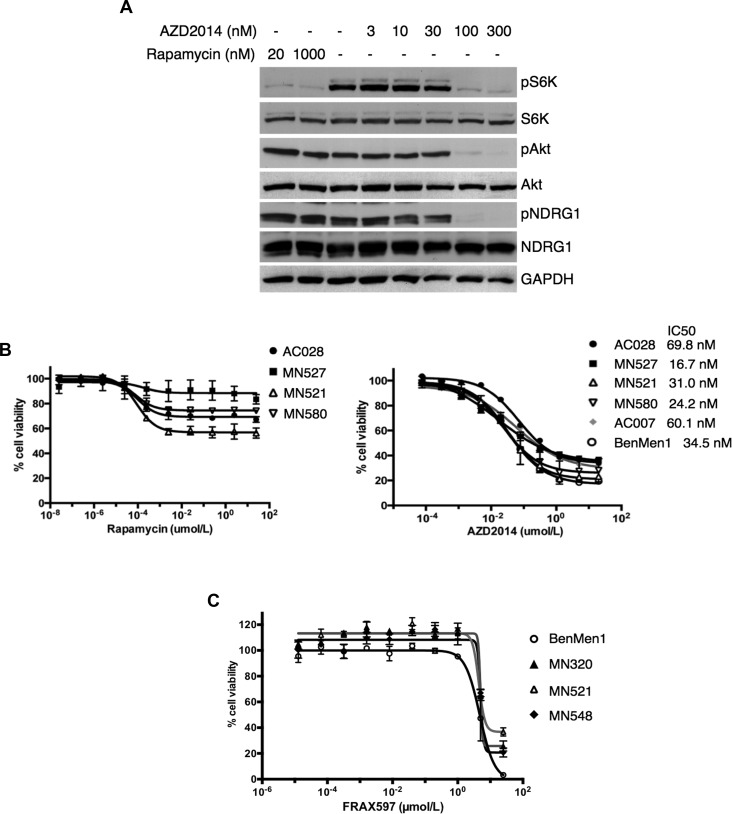
AZD2014 is more effective than rapamycin or FRAX597 in decreasing cell viability in NF2-deficient primary meningioma cells **A.** Immunoblotting of a patient-derived, NF2-deficient primary meningioma cell line reveal attenuation of pS6K (mTORC1 readout) upon treatment with rapamycin (20 and 1000 nM, 1h; lanes 1 and 2) with no change in pAkt or pNDRG1 (mTORC2 readouts) compared to DMSO alone (lane 3). In contrast, treatment with AZD2014 (3-300 nM, 1h; lanes 4-8) shows dose-dependent inhibition of both mTORC1 activity (pS6K) and mTORC2 signaling (pAkt and pNDRG1). S6K, Akt, NDRG1 and GAPDH serve as controls. **B** and **C.** Dose-response curves for AZD2014 and rapamycin were determined for NF2-deficient primary (MN521, MN527, MN580) and immortalized (Ben-Men-1) meningioma cells lines, as well as primary (AC028) and immortalized arachnoid (AC007) cell lines, and dose-response curves for FRAX597 were carried out in NF2-deficient primary (MN320, MN521, MN548) and Ben-Men-1 meningioma cells lines as indicated. Cells were exposed to increasing concentrations of rapamycin (B, left panel) in a 10 point, 10-fold serial dilution series (0 - 25 μM); AZD2014 (B, right panel) in a 10 point, 4-fold serial dilution series (0 - 20μM); or FRAX597 **C.** in a 10 point, 5-fold serial dilution series (0 - 25 μM) for 72h. Cell viabilities were assessed using CellTiter-Glo assays and plotted as % relative to DMSO controls. Cell viability and IC_50_ measurements were performed in at least three independent experiments with similar results. Data are presented as mean +/− SD for 3 replicates/drug dosage point **B.**, or 4 replicates/drug dosage point **C.**.

We compared the effectiveness of AZD2014 to rapamycin in blocking activation of mTORC1 and mTORC2-SGK1 in WHO grades I and II primary MN cell lines lacking NF2 expression under full-serum growth conditions. A representative immunoblot of a grade II MN is shown in Figure 7A. AZD2014 inhibited phosphorylation of p70S6K (pS6K, mTORC1 readout), pAkt and SGK1/pNDRG1 (mTORC2 readouts) in a dose-dependent manner. AZD2014 efficiently inhibited both mTORC1-dependent and mTORC2-dependent substrate phosphorylation at 100 nM. As expected, 20 nM rapamycin inhibited mTORC1-dependent S6K phosphorylation, however, failed to inhibit mTORC2-dependent phosphorylation of NDRG1 even at 10-fold higher doses (1000 nM) than AZD2014. These data suggest that AZD2014 is superior to rapamycin in inhibiting mTOR signaling in NF2-deficient MN cells *in vitro*. To test whether growth of NF2-associated MNs may be dependent on increased mTORC1/mTORC2 signaling, we performed cell viability assays of seven independent NF2-deficient primary MN cells in the presence of either rapamycin or AZD2014. Data from three independent cell lines are shown. Cell viability/proliferation of NF2-deficient MNs was effectively inhibited by AZD2014 with IC_50_ values < 100 nM after 72 hours of treatment (Figure 7B, right). In contrast, rapamycin treatment was not as effective as AZD2014 at suppressing NF2-deficient meningioma cell proliferation. Although partial growth inhibition was observed with low doses of rapamycin (1 nM, Figure 7B, left) no further inhibition of cell viability/proliferation was observed with increasing the concentrations of rapamycin. These data indicate that AZD2014 has a much greater effect at inhibiting cell proliferation of NF2-deficient MN cells than rapamycin. Cell viability assays were also carried out with the group I PAK inhibitor FRAX597, which was effective in blocking the viability/proliferation of NF2-deficient human MN cells, but at higher concentrations (IC_50_ > 4 μM) that may not be suitable for treatment (Figure 7C).

## DISCUSSION

**Figure 8:**
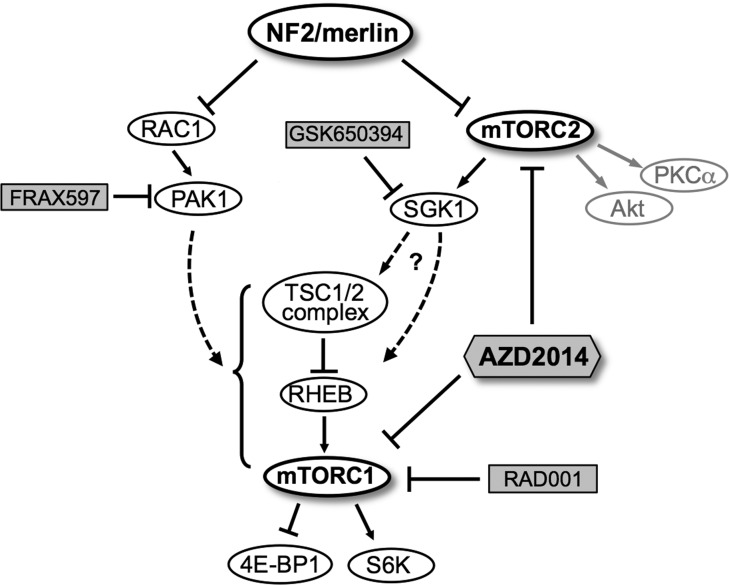
A schematic model for regulation of mTORC1/mTORC2 signaling pathways by NF2/merlin Schematic shows NF2 protein merlin as a negative regulator of RAC1-PAK1 signaling as well as a distinct mTORC2-SGK1 signaling axis independent of mTORC2-Akt. Upon loss of NF2, constitutive activation of mTORC2-SGK1 and/or RAC1-PAK1 pathways in turn may lead to aberrant mTORC1 activation. Treatment with rapamycin analogs such as RAD001 (mTORC1-specific) or group I PAK inhibitors (FRAX597) specifically inhibits mTORC1 signaling, whereas treatment with SGK1 inhibitor (GSK650394) or ATP-competitive mTOR kinase inhibitor (AZD2014) downregulates both mTORC2-SGK1 and mTORC1 signaling pathways. Chemical inhibitors are shaded. Dashed lines, potential pathways.

mTOR kinase forms two distinct functional complexes, mTORC1 and mTORC2, which signal to distinct downstream targets. mTORC1 phosphorylates p70S6K and 4EBP1 and is inhibited by rapamycin and its analogs such as RAD001 through an allosteric mechanism. mTORC2 phosphorylates Akt, PKC-α and SGK1 and is inhibited by mTOR kinase inhibitors that are ATP-competitive, but not by Rapalogs [14, 32]. Others and we have previously reported activation of mTORC1 in NF2-deficient human cells, leading to clinical trials with a rapamycin analog [7, 33]. Employing a high-throughput shRNA kinome screening, here we have identified candidate kinases that may contribute to the observed mTORC1 activation in NF2-deficient meningioma cells. In particular, we demonstrate that independent activation of SGK1 and PAK1 may be partly responsible for the mTORC1 activation in NF2-deficient meningioma cells (Figure 8, model). We further show elevated expression of SGK1 accompanied by constitutive activation of SGK1 as detected by phosphorylation of its specific target NDRG1, in human arachnoidal and meningioma cells with NF2 loss. Our results convincingly show that activation of the mTORC2-target SGK1/NDRG1 in human meningioma cells is sensitive to AZD2014, but insensitive to rapamycin. Treatment of primary meningioma cells with the dual mTORC1/mTORC2 inhibitor, AZD2014, leads to more profound suppression of cell proliferation when compared with rapamycin. Further studies are necessary to understand whether cell death mechanisms [34] are induced upon treatment with AZD2014.

Akt and SGK1 are AGC kinases downstream of mTORC2 that share approximately 50% identity [35]. Akt is generally considered as the critical effector of PI3K signaling, and most work in the cancer field is focused on Akt being the important mediator of cell proliferation upon PI3K activation. However, similar to Akt, SGK family members 1) are activated upon dual phosphorylation by PDK1 and mTORC2 in the PI3K pathway, 2) reveal overlapping substrate specificity with Akt, and 3) are implicated as potential players in malignant transformation [36-38]. In addition to Akt, phosphorylation of the SGK1-specific substrate NDRG1 is considered a reliable marker for mTORC2 activation [17, 39]. Interestingly, in NF2-null AC-CRISPR cells, upon deprivation of serum/growth factors, SGK1 is activated while Akt is not activated. Further, Akt inhibitor has no effect on either pS6 or pNDRG1 (Figure 5C). Taken together, it is likely that a distinct mTORC2-SGK1 signaling axis, independent of mTORC2-Akt, could be a major driver of proliferation in NF2-null AC and MN cells (Figure 8, model). A recent study documents increased SGK1 expression in a subset of breast cancer cell lines that are resistant to Akt inhibition, and treatment of these cells with an ATP-competitive mTOR kinase inhibitor leads to inhibition of SGK1 activation and suppression of proliferation, thus highlighting the role of SGK1 activation in breast cancer [38]. Further, in glioblastomas with activating mutation in EGFR, NF-κB is activated through mTORC2 in an SGK1-dependent manner that does not require Akt or mTORC1, underlying the Akt-independence of this pathway [40]. Taken together, it is important to examine the activation of SGK1/NDRG1 in addition to Akt when evaluating mTORC2 activation.

While much information is known about the regulation of mTORC1, the mechanism by which mTORC2 is regulated is poorly understood. We have observed constitutive activation of mTORC2-SGK1 in NF2-null ACs upon serum/growth factor deprivation, and further studies are necessary to understand the mechanism of this activation. One possibility is the elevated level of several growth factor receptors, including ERBB3, observed upon NF2 loss at the plasma membrane [41, 42] that may feed into and activate the mTOR pathway. It is also possible that GRK7, EPHA5, and/or ERBB3 that we have identified in the kinome screen (Table 1) may play a role upstream of mTORC1/mTORC2, and further studies are essential on these and the other validated hits from the screen. Our work also raises the question of how mTORC2 distinctly activates SGK1 independent of Akt under growth factor-deprived conditions in NF2-null cells. A physical association between the mTORC2-specific component, mSIN1 (mammalian stress-activated protein kinase-interacting protein 1) and SGK1 is shown to be important for the mTORC2-mediated phosphorylation of SGK1 and its activation, which appears to be distinct from Akt, since Akt does not bind to mSIN1, raising the possibility that mTORC2 could selectively associate with its substrates and regulate specific cellular process [43]. Based on the increased expression of SGK1 that we observe upon NF2 loss, it is tempting to speculate whether enhanced binding between SGK1 and mSIN1 occurs in NF2-deficient cells, which could be responsible for the selective activation of SGK1. Taken together it is possible that upstream signaling that activates an mTORC2-SGK1 axis may be context-specific and not necessarily shared with an mTORC2-Akt axis.

To date, there are no medical therapies that have proven efficacious for meningiomas, either sporadic or NF2-related. Moreover, there are no relevant genetic mouse models for drug testing in meningiomas, especially for recurrent or progressive tumors. *Nf2* mutant mice (*Nf2*^+/−^) do not develop schwannomas or meningiomas, and selective Cre-mediated excision of *Nf2* in arachnoidal cells results in development of benign (grade I) meningioma in mice [44]. However, only 30% of these mice develop intracranial meningiomas, which are microscopic and require a prolonged time to appear, thus limiting the use of this *in vivo* model for therapeutic studies. Our earlier work in human meningioma cells demonstrating the activation of mTORC1 led to clinical trials with rapamycin analogs for NF2 and sporadic meningioma patients. Rapamycin is merely cytostatic in our cells, consistent with the clinical outcome thus far observed with RAD001/everolimus. Moreover, rapamycin treatment is known to relieve the negative feedback inhibition on IRS-1 [45, 46], Grb10 [47, 48] as well as other negative regulation of mTORC2 independent of IRS-1 and Grb10 [49, 50], and thus can activate PI3K/Akt and ERK/MAPK prosurvival pathways. Similarly, here we have shown that rapamycin treatment can also upregulate mTORC2-SGK1 signaling (Figure 5D). As an AGC kinase family member closely related to Akt, SGK1 can also phosphorylate Akt substrates such as FOXO3a and BAD and lead to potential survival signals [36, 37]. Here we establish that, in addition to mTORC1 activation, mTORC2-dependent activation of SGK1/NDRG1 is commonly seen in human meningioma cells, which remains unaffected by rapamycin treatment. Our studies indicate that the selective mTOR kinase inhibitor AZD2014, which targets both mTORC1 and mTORC2, is more efficient than mTORC1 inhibitor rapamycin in blocking proliferation of primary human meningioma cells. Importantly, AZD2014 is currently in use in AstraZeneca-sponsored clinical trials as well as other ongoing Externally Sponsored Research (ESR) studies for patients with many types of cancer [51]. Therefore, we believe that there is a timely opportunity for testing the ATP-competitive dual mTORC1/mTORC2 inhibitor AZD2014, which may offer critical therapeutic advantage over rapalogs for recurrent or progressive meningiomas.

## MATERIALS AND METHODS

### Cell lines and reagents

Cell lines included immortalized NF2-deficient meningioma Ben-Men-1 [15], immortalized arachnoid AC007-hTERT derived from an NF2 patient expressing heterozygous level of NF2 [7, 8], primary normal arachnoid AC028, and several primary NF2-deficient meningioma (MN) lines. Ben-Men-1 cells and AC007-hTERT were maintained under growth conditions described [7, 15], and all primary lines were established and maintained as reported [8]. Low passage 293T cells for large-scale lentiviral packaging were obtained from TRC. Insulin was from Sigma (St. Louis, MO). Inhibitor reagents included rapamycin and AktVIII (EMD Millipore; Billerica, MA), AZD2014 (provided by AstraZeneca; Wilmington, DE), GSK650394 (Tocris; Minneapolis, MN), Torin1 (kindly provided by Dr. David Sabatini, Whitehead Institute/MIT, Cambridge, MA) and FRAX597 (generously given by Dr. Joseph Kissil, The Scripps Research Institute, Jupiter, FL). Inhibitor treatment times and concentrations are described in figure legends. All inhibitors were diluted as per manufacturer's recommendations.

### High-throughput shRNA screen

The immunofluorescence-based kinome screen was carried out using Ben-Men-1 in a 384-well plate format. The entire kinome library, developed by TRC, consisted of high titer lentivirus for 6,091 shRNAs, cloned into the pLKO-puromycin system, targeting 747 distinct kinases that were arrayed on 18 384-well plates (5-10 shRNAs/kinase). Each library virus plate contained internal negative controls including shRNAs targeting irrelevant reporter genes (*GFP, RFP, luciferase* and *LacZ*), a non-hairpin forming (nullT) control and media alone (no virus). Each screening plate included a positive control lentiviral human *RHEB*-shRNA (TRCN0000010424). In addition, a separate library control plate was used consisting of 95 distinct negative control hairpins. Infections included 4 biological replicates, 3 under puromycin (puro) selection (0.75μg/ml, Life Technologies; Grand Island, NY) and 1 without puro. All subsequent steps (addition of lentivirus to fixation) were carried out by TRC using robotic liquid handling. At 18-20h after plating, each shRNA lentivirus was transferred onto cells in the presence of 5μg/ml polybrene (Sigma). The cells were spun at 2250rpm at 37°C for 30min, and transferred to 37°C overnight. The following day, virus was removed and fresh growth media was added (with or without puro). After 72h, cells were incubated in serum free media for 18-20h and fixed in 4% paraformaldehyde (Electron Microscopy Sciences; Hatfield, PA) at room temperature for 20min. Cells were permeabilized and blocked in one step with 0.2% Triton X-100/2%BSA/PBS for 20min at room temperature followed by co-incubation with AlexaFlour488 conjugated-pS6 (Ser240/244) antibody (Cell Signaling Technology; Beverly, MA) and DAPI (Life Technologies) for 18-20h at +4°C. Analysis for pS6 staining intensity was performed using the Acumen eX3 laser-scanning fluorescence microplate cytometer. Immunofluorescent staining in Figure 1 was visualized on a Nikon (Tokyo, Japan) Eclipse TE2000-U inverted microscope using the EXFO X-Cite 120 fluorescent illumination system. Images were acquired with a Nikon DS-QiMc camera and NIS-Elements BR imaging software.

### Large-scale lentiviral packaging and infection

For large-scale packaging, pLKO-puro-based shRNAs targeting human *SGK1* (TRCN0000194957), human *PAK1* (TRCN0000197238), human *RHEB* (TRCN0000010424) as well as pLKO.1-emptyT (nullT) control (TRCN0000208001) were obtained from TRC, and packaged using TRC methods (http://www.broadinstitute.org/rnai/public/resources/protocols). For viral infection, Ben-Men-1 cells were transduced using lentiviral shRNAs for each of the following hairpins: SGK1, PAK1, RHEB, nullT control, as well as no virus control. Spin-infection was carried out at 2000rpm for 1.5h at 37°C, and then cells were transferred to 37°C overnight. The following day, cells were passaged to a 15cm plate, followed by puro selection (0.5μg/ml) for 3 days with no live cells observed in the control without virus. Media was exchanged for fresh growth media without puro prior to all further experiments.

### Immunoblotting and antibodies

SDS-PAGE and immunoblotting were carried out as described [7]. Antibodies for phospho-S6 (S240/244), S6, phospho-p70 S6K (T389), p70 S6K, phospho-Akt (S473), Akt, phospho-NDRG1 (T346), SGK1, phospho-PAK1 (S144), PAK1, and 4EBP1 were from Cell Signaling Technology. Other antibodies included NDRG1 (Abcam; Cambridge, MA), Rac1 (BD Biosciences; San Jose, CA), GAPDH (EMD Millipore), Rheb (3M6 monoclonal; a generous gift from Richard Lamb, University of Liverpool, U.K.) and the NF2 polyclonal C26 antibody has been described [52].

### Generation of isogenic human arachnoidal cell lines using CRISPR-Cas9 gene editing

To generate isogenic NF2-expressing and NF2-deficient human arachnoidal cells (ACs), we used the AC007-hTERT cell line (AC cells). AC cells were transfected using the basic primary fibroblasts Nucleofector kit and the AmaxaNucleofector II (Lonza; Walkersville, MD) according to the manufacturer's instructions. Briefly, AC cells were resuspended in Nucleofector solution, and then transfected using program U-023 with lenti-CRISPR-NF2_sg1 expressing a single guide RNA targeting human *NF2* exon 8, the Cas9 enzyme, and a puro selection cassette (a kind gift from the Zhang laboratory at the Broad Institute and MIT [28]). Following transfection, cells were re-plated sparsely to form single colonies. Single AC-CRISPR cell clones were picked and expanded for genomic DNA extraction, PCR of human *NF2* exon 8, and Sanger sequencing to confirm in/del mutations. PCR primers: *hNF2* ex 8-F, 5′-GGGACCCAGAAGTC ACAAGA-3′; *hNF2* ex 8-R, 5′-TTTCATTATGCATGCCCAGA-3′.

### RNA isolation, cDNA synthesis and quantitative PCR

AC-CRISPR cells were lysed in TRIzol Reagent (Life Technologies), rinsed in chloroform, and aqueous layer was applied to Qiagen RNeasy kit (Qiagen; Santa Clarita, CA) columns followed by RNA purification according to manufacturer's instructions. For cDNA synthesis, the Superscript VILO cDNA synthesis kit (Life Technologies) was used according to the manufacturer's instructions, and quantitative RT-PCR (q-RT-PCR) was carried out in triplicate on a Roche (Indianapolis, IN) Lightcycler 480 (software version 1.5.0 SP3) using iQ^−^SYBR Green Supermix (Bio-Rad; Hercules, CA). For human *SGK1*, two independent primer sets were used including primer set #1 (254bp amplicon): *hSGK1* qPCR-1F, 5′-ATGACGGTGAAAACTGAGGCT-3′; *hSGK1* qPCR-1R, 5′-GTTCTCCTTGCAGAGTCCGAAG-3′, which were validated by TRC/Broad Institute (primer sequences from PrimerBank, http://pga.mgh.harvard.edu/primerbank/index.html) and primer set #2 (132bp amplicon): *hSGK1* qPCR-2F, 5′-GCTGAAATAGCCAGTGCCTTGG-3′; *hSGK1* qPCR-2R, 5′-GTTCTCCTTGCAGAGTCCGAAG-3′. Human 18S served as a control using primers: *h18S*-F, 5′-ACCCGTTGAACCCCATTCGTGA-3′; *h18S*-R, 5′-GCCTCACTAAACCATCCAATCGG-3′. Melt curves showed single peak specificity for each q-RT-PCR primer set.

### Statistical analysis

For the high-throughput screen, data acquisition, analysis and top hit calling was performed by TRC using robust z scoring methodology to normalize data [16]. For q-RT-PCR, all experiments were performed in triplicate. Fold changes in gene expression were calculated using the comparative *CT* (threshold cycle) method, and expression levels were quantitated relative to control (normalized to 1.0). Data values are represented as mean +/− SD. Within each group, student *t*-test was performed with a value of *p* < 0.05 considered significant.

### Cell viability assays

Cell viability was assessed in 384-well plates using CellTiter-Glo Luminescent Cell Viability Assay (Promega; Madison, WI). Briefly, the next day after seeding, cells were treated with serial dilutions of AZD2014, rapamycin or FRAX597 in full growth media and incubated for 72h (see figure legends for dilution point details). DMSO was used as a control. Luminescence was detected using an EnVision Plate Reader (Perkin Elmer; Waltham, MA) and mean values +/− SD from at least 3 independent experiments were determined for each meningioma cell line. Growth curves were plotted using GraphPad Prism 6.0 software (San Diego, CA) and drug concentrations inhibiting cell growth by 50% (IC_50_) was determined using nonlinear regression (curve fit) analysis.
